# Reading the fine print: A closer look at the Arabidopsis ORFeome

**DOI:** 10.1093/plcell/koad293

**Published:** 2023-11-28

**Authors:** Michael Busche

**Affiliations:** Assistant Features Editor, The Plant Cell, American Society of Plant Biologists; Laboratory of Genetics, University of Wisconsin, Madison, WI 53706, USA

Translation is a complex and highly regulated cellular process by which proteins are produced using RNA molecules as templates. It is carried out by ribosomes in conjunction with accessory proteins, including initiation, elongation, and termination factors, as well as transfer RNAs and transfer RNA synthetases. In theory, this process can occur on any RNA molecule with an open reading frame (ORF), a sequence of nucleotides that begins with a start codon and ends with a stop codon. Reliably identifying and annotating ORFs is an important first step in uncovering the biological function of the molecules they encode.

Defining an ORF relies on obtaining high-quality sequence data. As such, short ORF sequences are relatively harder to identify than those in longer or highly expressed transcripts. Additionally, current computational methods for annotating ORFs often have a bias against small ORFs to minimize the misidentification of true ORF sequences. **Hsin-Yen Larry Wu and colleagues** (Wu et al. 2023) have recently made important discoveries in this understudied area of the translational landscape in Arabidopsis. In this work, the authors demonstrate how they used an improved ribosome profiling technique, together with modified computational approaches, to identify over 7,000 previously unannotated translational events (Fig. 1).

**Figure 1.**
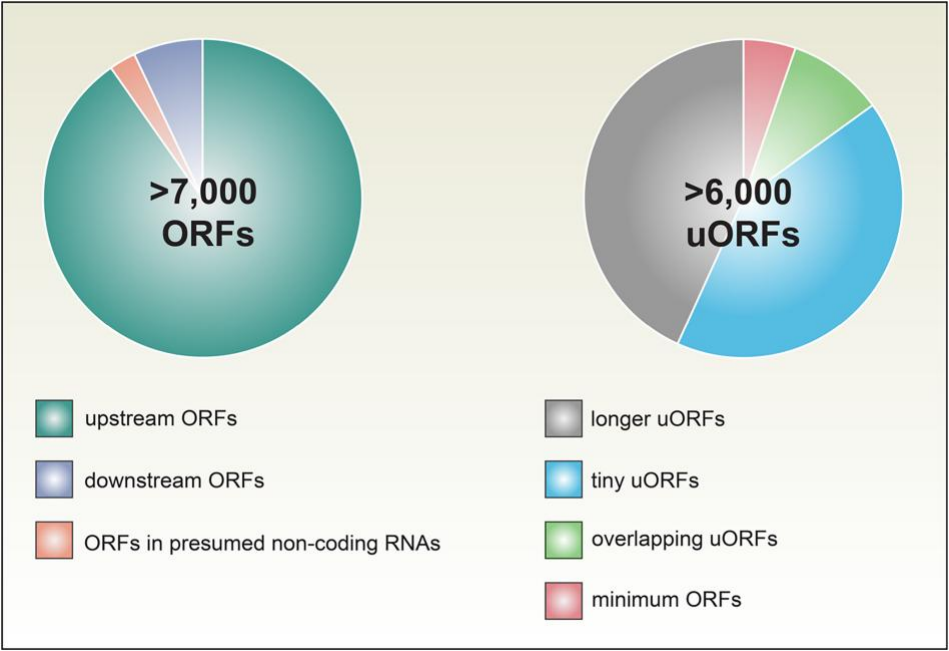
ORFs vary in their size and position. Wu et al. (2023) used improved ribosome profiling and computational filtering strategies to identify 7,751 unconventional translation events. These included 6,996 ORFs upstream of annotated protein-coding genes (uORFs), 209 ORFs downstream of such genes, and 546 ORFs within presumed noncoding RNAs. Of the uORFs identified, 681 overlapped with another ORF; 2,921 encoded peptides only 2 to 10 amino acids in length (tiny uORFs); and 370 were the minimum length of start codon followed by a stop codon (minimum ORFs). Figure designed by M. Busche using Adobe Illustrator.

Ribosome profiling, also known as ribosome footprinting or Ribo-seq, is a technique that allows scientists to determine which specific RNAs are being translated. When paired with traditional RNA sequencing, it can be used to calculate the translation efficiency of specific transcripts. Wu et al. used this technique to identify small ORFs, noncoding ORFs, and ORFs located upstream (uORFs) or downstream of “main” protein-coding ORFs (mORFs) within the Arabidopsis nuclear, chloroplast, and mitochondrial genomes. These data even included minimum uORFs that consist of a start codon followed by a stop codon, as well as tiny uORFs that encode peptides of just 2 to 10 amino acids.

Wu et al. continued their research by characterizing the effects of uORFs on the translation of adjacent mORFs. The researchers compared wild-type uORF sequences with mutated versions lacking the start codon. By inserting these upstream of genes encoding the reporter enzyme luciferase, they observed significant changes in translational output (i.e. luciferase levels) in response to the uORF sequence. In fact, even the presence of minimum and tiny uORFs decreased the translation efficiency of corresponding downstream mORFs.

The authors went on to explore the position, sequence, conservation, evolution, and function of the novel ORFs they identified. Their data revealed striking differences in translation between mitochondrial and nuclear ORFs, helped in identifying ORFs on tasiRNAs, and more. This work not only expands our current understanding of translation and its regulation in Arabidopsis but also introduces several new tools for investigating aspects of this biological process that were previously difficult to study.

## References

[koad293-B1] Wu HL , Ai Q, Teixeira RT, Nguyen PHT, Song G, Montes C, Elmore JM, Walley JW, Hsu PY. Improved super-resolution ribosome profiling reveals prevalent 5′ translation of upstream ORFs and small ORFs in Arabidopsis. Plant Cell. 2023. 10.1093/plcell/koad197 PMC10896292 38000896

